# Prioritization of Cancer Marker Candidates Based on the Immunohistochemistry Staining Images Deposited in the Human Protein Atlas

**DOI:** 10.1371/journal.pone.0081079

**Published:** 2013-11-26

**Authors:** Su-Chien Chiang, Chia-Li Han, Kun-Hsing Yu, Yu-Ju Chen, Kun-Pin Wu

**Affiliations:** 1 Institute of Biomedical Informatics, National Yang Ming University, Taipei, Taiwan; 2 Institute of Chemistry, Academia Sinica, Taipei, Taiwan; 3 College of Medicine, National Taiwan University, Taipei, Taiwan; National Central University, Taiwan

## Abstract

Cancer marker discovery is an emerging topic in high-throughput quantitative proteomics. However, the omics technology usually generates a long list of marker candidates that requires a labor-intensive filtering process in order to screen for potentially useful markers. Specifically, various parameters, such as the level of overexpression of the marker in the cancer type of interest, which is related to sensitivity, and the specificity of the marker among cancer groups, are the most critical considerations. Protein expression profiling on the basis of immunohistochemistry (IHC) staining images is a technique commonly used during such filtering procedures. To systematically investigate the protein expression in different cancer versus normal tissues and cell types, the Human Protein Atlas is a most comprehensive resource because it includes millions of high-resolution IHC images with expert-curated annotations. To facilitate the filtering of potential biomarker candidates from large-scale omics datasets, in this study we have proposed a scoring approach for quantifying IHC annotation of paired cancerous/normal tissues and cancerous/normal cell types. We have comprehensively calculated the scores of all the 17219 tested antibodies deposited in the Human Protein Atlas based on their accumulated IHC images and obtained 457110 scores covering 20 different types of cancers. Statistical tests demonstrate the ability of the proposed scoring approach to prioritize cancer-specific proteins. Top 100 potential marker candidates were prioritized for the 20 cancer types with statistical significance. In addition, a model study was carried out of 1482 membrane proteins identified from a quantitative comparison of paired cancerous and adjacent normal tissues from patients with colorectal cancer (CRC). The proposed scoring approach demonstrated successful prioritization and identified four CRC markers, including two of the most widely used, namely CEACAM5 and CEACAM6. These results demonstrate the potential of this scoring approach in terms of cancer marker discovery and development. All the calculated scores are available at http://bal.ym.edu.tw/hpa/.

## Introduction

Quantitative proteomics has been used widely in cancer marker discovery with a certain degree of success [1]–[7]. This type of study usually generates a huge amount of data that need to be further analyzed in order to identify marker candidates. Although there is no standard way to screen cancer markers from massive proteomic datasets [8], these efforts have delivered a number of potential cancer markers [9]–[11]. Even though various approaches have been developed, mining biomarkers from high-throughput proteomic data primarily relies on fold changes in protein expression between the normal and cancer groups [12]. A good cancer marker is expected to be highly overexpressed in the appropriate cancer group, and the degree of the overexpression needs to be both significant and specific to the cancer of interest.

A method that is able to define the cancer-specificity of a protein to the cancer of interest is therefore indispensible. To create such a cancer-specificity index, we need to have expression information on the various proteins in healthy individuals and in patients with different types of cancer. Acquiring such proteomic data, however, is resource and time-consuming for small-scale academic research groups. Fortunately the Human Protein Atlas (HPA) is available; this comprehensively annotates a large number of genes and proteins expressed in various types of normal and cancer tissues [13]–[15]. HPA is an antibody-based database. By applying tissue microarray and immunohistochemistry (IHC) staining techniques, HPA has comprehensively accumulated millions of high-resolution images with expert-curated annotations. IHC staining is regarded as an effective technique in proteomic research [16], [17]. On the basis of these images, especially those using IHC staining, the HPA has been effectively used in a number of studies for cancer marker discovery [18]–[24]. The approach used with the HPA in these studies, however, involved manual queries. Since the annotation of the IHC images is ordinal and denoted by gradient bars, acquiring protein expression levels from the HPA is unintuitive and labor-intensive. Moreover, when examining the gradient bars of the IHC annotations, subjective judgment comes into play and this may make interpretation of protein expression level by the researchers inconsistent across different images. Accordingly, a systematic way to quantify protein expression data from the HPA, which would allow the cancer specificity of proteins to be defined on the basis of the IHC annotations of HPA, becomes essential.

In this study, we proposed a scoring approach based on the annotation of the IHC images from the HPA. The scoring approach takes into account a protein's expression levels in normal/cancer tissues and the significance/specificity of any overexpression of the protein in the cancer tissue. On the basis of the proposed scoring mechanism, we comprehensively prioritized all the tested antibodies in the HPA (17219 antibodies in the HPA version 10.0) for 20 different types of cancers. A statistical analysis of the results was carried out by the one-sample *t*-test and this demonstrated that the proposed scoring approach is able to identify proteins that are overexpressed in cancer tissues, and pinpoint when such overexpression is significant and specific to the cancer of interest. We also used a sample cohort of 1482 proteins [25] to evaluate the effectiveness of the proposed scoring approach. The scoring approach, in combination with protein fold changes, was able to identify four marker candidates for colorectal cancer from the sample cohort. The four selected marker candidates included CEACAM 5 and CEACAM6, which are the most widely used markers for colorectal cancer at present; they are primarily used for prognostic monitoring [26]. The other two selected marker candidates, CAMP and ANXA4, have also been reported to be potential markers for colorectal cancer [27]–[29]. The evaluation results demonstrate the potential of the proposed scoring approach when it is applied to cancer marker discovery. All the calculated scores are available for query via a web site, “HPA Scoring” at http://bal.ym.edu.tw/hpa/.

## Materials and Methods

### The IHC images of HPA

In this study, immunohistochemistry (IHC) staining images of the HPA version 10.0 released on the 12 September 2012 (http://www.proteinatlas.org/) were used to prioritize genes or proteins represented by antibodies. Data entries in the HPA are indexed using their gene names. In the HPA version 10.0, there are 14012 genes, the protein expression profiles of which are measured using 17219 antibodies in 46 normal human tissue types, 20 cancer tissue types, and 47 human cell lines. HPA version 10.0 has comprehensively accumulated millions of high-resolution IHC images with expert-curated annotations, among which 5108055 were used in this study.

### Validation dataset

A cohort of 1482 membrane proteins expressed in paired tumor and adjacent normal tissues from 28 patients diagnosed with colorectal cancer was used as our validation dataset [25] (Table S1). Clinical information on the 28 patients is presented in Table S2. This dataset was originally created to screen potential markers for colorectal cancer.

### Mapping the cancer and normal tissues

The proposed scoring approach is primarily based on using protein expression differences between cancer and normal tissues. Therefore there was a need to map the relationship between the various types of cancer and their paired normal tissues. These mappings, which were extracted from the HPA, are listed in Table 1. A cancer type may be defined in a number of different mappings if it is either paired with more than one cell type in a normal tissue (e.g. cervical cancer is paired with glandular cell and squamous epithelial cell from cervix, uterine) or paired with more than one normal tissue type (e.g. colorectal cancer is paired with tissue from the colon and rectum). The different mappings are analyzed independently when our approach is applied. Please note that there is no mapping defined for ovarian cancer due to a lack of IHC staining results in the HPA for normal ovary tissue. Furthermore, since hepatocellular carcinoma and cholangiocarcinoma are totally different cancers, they were regarded as different cancer types in our mappings even if they were all classified as liver cancer in the HPA. Eventually, 27 mappings were defined for 20 cancer types using the HPA. Please note that we did not investigate cancer subtypes, such as lobular carcinoma and duct carcinoma, which are breast cancers, because in such cases the number of tissue samples in the HPA is quite limited. Our approach is antibody-oriented; each antibody in the HPA is used to evaluate no more than 12 patients with a certain type of cancer. If we further classify the corresponding 12 IHC images into different cancer subtypes, it would be very difficult to draw any conclusion from statistical significant evidence that is based solely on <10 IHC images. We would like to emphasize that looking into cancer subtypes is a very important aspect of cancer marker discovery. We will make our effort towards this direction when the HPA or another database is able to provide a sufficient number of IHC images of different cancer subtypes.

**Table 1:** Mappings between cancer tissues and normal tissues.

Cancer	Normal Tissue (Cell Type)	Mapping ID
Breast cancer	breast (glandular cells)	Breast
Carcinoid	pancreas (islets of Langerhans)	Carcinoid
Cervical cancer	cervix, uterine (glandular cells)	Cervical-A
	cervix, uterine (squamous epithelial cells)	Cervical-B
Colorectal cancer	colon (glandular cells)	Colorectal-A
	rectum (glandular cells)	Colorectal-B
Endometrial cancer	uterus, pre-menopause (glandular cells)	Endometrial-A
	uterus, post-menopause (glandular cells)	Endometrial-B
Glioma	cerebral cortex (glial cells)	Glioma
Head and neck cancer	oral mucosa (squamous epithelial cells)	Head & neck-A
	salivary gland (glandular cells)	Head & neck-B
Cholangiocarcinoma	liver (bile duct cells)	Cholangio
Hepatocellular carcinoma	liver (hepatocytes)	Hepato
Lung cancer	bronchus (respiratory epithelial cells)	Lung-A
	lung (pneumocytes)	Lung-B
Lymphoma	lymph node (germinal center cells)	Lymphoma-A
	lymph node (non-germinal center cells)	Lymphoma-B
Melanoma	skin (melanocytes)	Melanoma
Ovarian cancer*	N/A	
Pancreatic cancer	pancreas (exocrine glandular cells)	Pancreatic
Prostate cancer	prostate (glandular cells)	Prostate
Renal cancer	kidney (cells in tubules)	Renal
Skin cancer	skin (keratinocytes)	Skin
Stomach cancer	stomach, lower (glandular cells)	Stomach-A
	stomach, upper (glandular cells)	Stomach-B
Testis cancer	testis (cells in seminiferus ducts)	Testis
Thyroid cancer	thyroid gland (glandular cells)	Thyroid
Urothelial cancer	urinary bladder (urothelial cells)	Urothelial

*Ovarian cancer was not available because most of the antibodies in HPA database were not evaluated against normal ovary tissues.

### Expression differences as detected by antibody in relation to mapped cancer and normal tissues

For a given mapping and a given antibody, our aim was to determine the expression difference (*ED*) of the target protein between the paired cancer and normal tissue samples. Expression levels of a protein in tissues are determined based on the annotations provided by the HPA. Each gene in the HPA is annotated; this consists of a gene and protein summary, antibody and antigen information, and a range of different types of expression profiles. In this study, the annotations *Intensity* and *Quantity* for IHC staining are used to define the expression level of a protein in tissues. The annotation *Intensity* represents the level of antibody staining. The annotation *Quantity* represents the fraction of positively stained cells. Since a protein may be recognized by more than one antibody due to multiple binding sites, certain genes in the HPA are evaluated using more than one antibody. Since antibodies used to create the HPA are not all of the same quality, the evaluation of the results from these antibodies may be inconsistent. To address this issue, our proposed approach is designed to be antibody-oriented in order to overcome any inconsistencies in the quality of antibody. Different antibodies for a given gene product are regarded as distinct data entries and processed separately.

**Figure 1:**
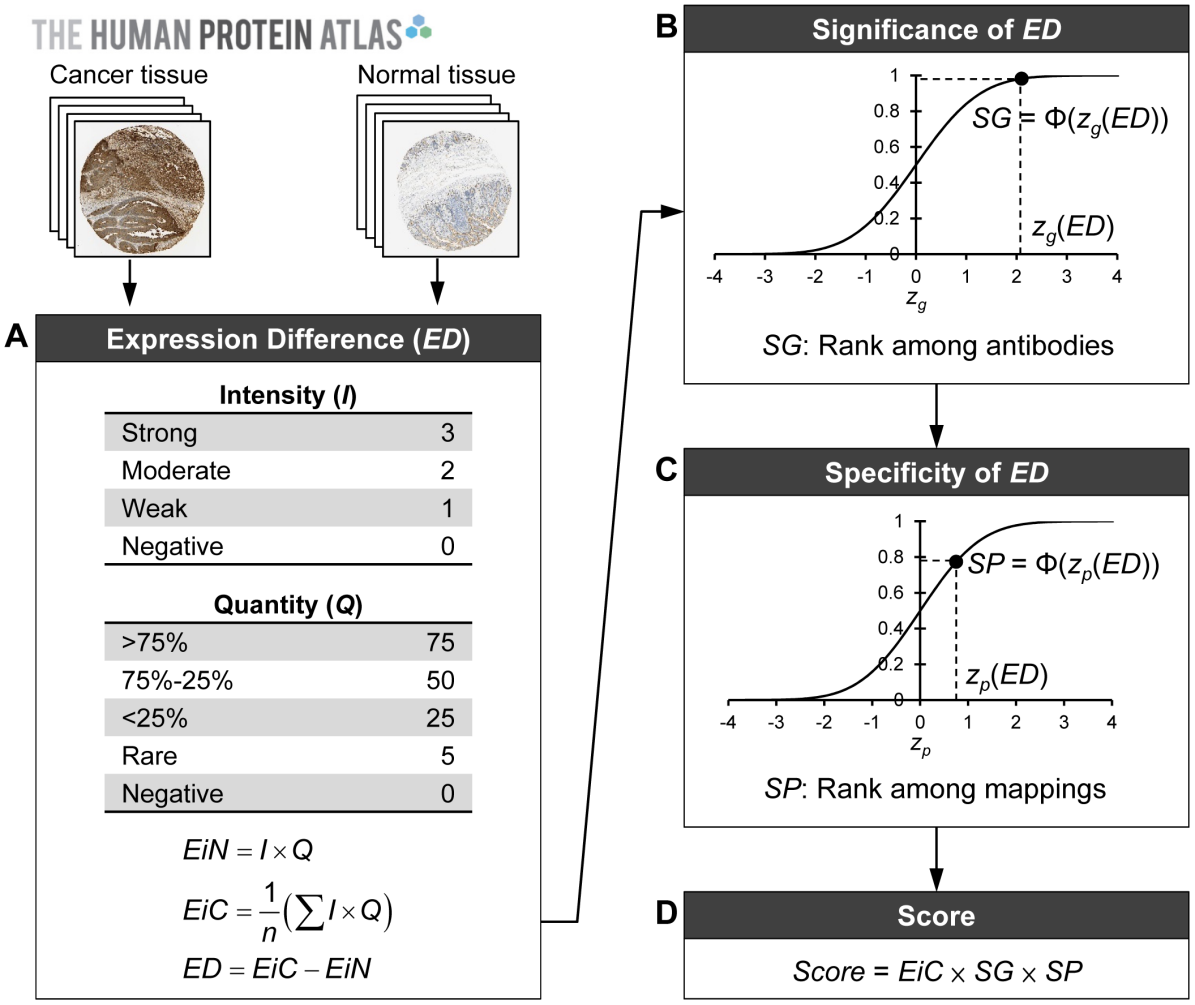
Procedure for determining the score of an antibody in relation to a mapping of interest. (A) Initially, the protein expression levels and the expression difference (*ED*) between cancer tissue and normal tissue for all antibodies covering all mappings are calculated. (B) The significance of the target *ED* with respect to the mapping of interest is determined by a cumulative z distribution. (C) The specificity of the target *ED* with respect to the mapping of interest is determined by another cumulative z distribution. (D) The final score of the antibody with respect to the mapping of interest is determined on the basis of its protein expression level in cancer tissue and the significance and specificity of its *ED*.

For the target protein, its expression in tissues is characterized by the annotations *Intensity* and *Quantity*. The two annotations are first transformed from ordinal form to numeric form. The four values Strong, Moderate, Weak, and Negative that are used to describe *Intensity* are transformed into 3, 2, 1, and 0, respectively. The transformed *Intensity* is denoted by *I*. Similarly, the five values >75%, 75%–25%, <25%, Rare, and Negative that are used to describe *Quantity* are transformed into 75, 50, 25, 5, and 0, respectively. The transformed *Quantity* is denoted by *Q*. The basic factor defining the expression of a protein in tissues is then calculated using *I*×*Q* (Figure 1A).

For the normal cell type, no matter how many times the antibody is used to perform the IHC staining, HPA only reports one pair of *Intensity* and *Quantity* scores. We therefore have only one pair of *I* and *Q* values for the normal cell type. The expression of the protein in the normal cell type, *EiN* (expression in normal), is therefore defined as follows: 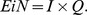
For example, there is only one pair of *Intensity* and *Quantity* (Moderate, >75%) when the antibody HPA034966 is used for the IHC staining of glandular cells from normal breast tissue, we therefore have *EiN*  = 2×75 = 150. Overall, the values of *EiN* will have a range from 0 to 225.

In contrast to the situation for normal tissue, for a given cancer type, the HPA reports a pair of *Intensity* and *Quantity* each time the antibody is used to perform IHC staining. Consequently, we usually have several pairs of *I* and *Q* values for a given cancer type. Thus the expression of a protein in a given cancer type, *EiC* (expression in cancer), is defined as the average expression of the protein in tissues from the patients diagnosed with this cancer:
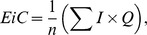
where *n* is the number of tested patients diagnosed with this cancer. For example, the antibody HPA034966 was used to perform IHC staining on 12 patients with breast cancer and as a result the HPA provides 12 pairs of *Intensity* and *Quantity* scores; these are: (Strong, >75%), (Moderate, >75%), (Strong, >75%), (Strong, >75%), (Moderate, >75%), (Moderate, >75%), (Moderate, >75%), (Moderate, >75%), (Moderate, >75%), (Moderate, >75%), (Moderate, >75%), and (Moderate, >75%). We therefore have *EiC*  = (3×75+2×75+3×75+3×75+2×75+2×75+2×75+2×75+2×75+2×75+2×75+2×75)/12 = 2025/12 = 168.75. Overall, the values of *EiC* will also have a range from 0 to 225.

Finally, the expression difference, *ED*, of a given antibody for a given mapping is defined as *ED*  =  *EiC*-*EiN* (Figure 1A).

### Antibody scores in relation to tissue mapping

For a given antibody and a given mapping, the antibody is expected to receive a high score if (1) the target protein is overexpressed in the cancer tissue, and (2) the degree of the overexpression is significant and specific to the mapping. The score of the antibody to the mapping is therefore determined using the following steps (Figure 1):

1. **Determine the protein expression and ED of all antibodies.** In the initial step, we first determine the protein expression levels *EiC* and *EiN* for all the antibodies in HPA for all mappings. The expression difference *ED* of antibodies is determined using *EiC*-*EiN* (Figure 1A). Please note that this initial step can be regarded as the “system initialization” and is performed only once; the calculated *EiC*'s, *EiN*'s, and *ED*'s remain constant for the scoring of all antibodies.
2. **Determine the significance of the target ED.** We would like to know if the *ED* of the target antibody is significant in relation to the mapping of interest. The *ED* values of all antibodies to this mapping are normalized by z-score transformation 
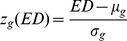
 to remove inter-experiment bias, where µ*_g_* and σ*_g_* are the mean and standard deviation of all these *ED*'s, respectively. The *significance* of the *ED* of the target antibody to the mapping, *SG*, is defined by the cumulative z distribution *SG*  =  *P*(*Z*≤ *z_g_*(*ED*)) (Figure 1B). *SG* can be regarded as the rank of the target antibody among all antibodies with respect to the mapping of interest. The value of an *SG* will be within the range from 0 to 1.
3. **Determine the specificity of the target ED.** We also wish to know if the target *ED* is specific to the mapping of interest. The *ED*'s of the target antibody to all mappings are normalized by z-score transformation 
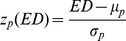
 to remove inter-experiment bias, where µ*_p_* and σ*_p_* are the mean and standard deviation of all these *ED*'s, respectively. The *specificity* of the *ED* of the target antibody to the mapping, *SP*, is defined by the cumulative z distribution *SP*  =  *P*(*Z*≤ *z_p_*(*ED*)) (Figure 1C). *SP* can be regarded as the rank of the target mapping among all mappings with respective to the target antibody. The value of an *SP* will also be within the range from 0 to 1.
4. **Determine the score of the target antibody.** The score of a given target antibody in relation to a given mapping of interest is defined as 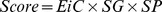 (Figure 1D). The value of a *Score* will be within the range from 0 to 225.

## Results and Discussion

**Figure 2:**
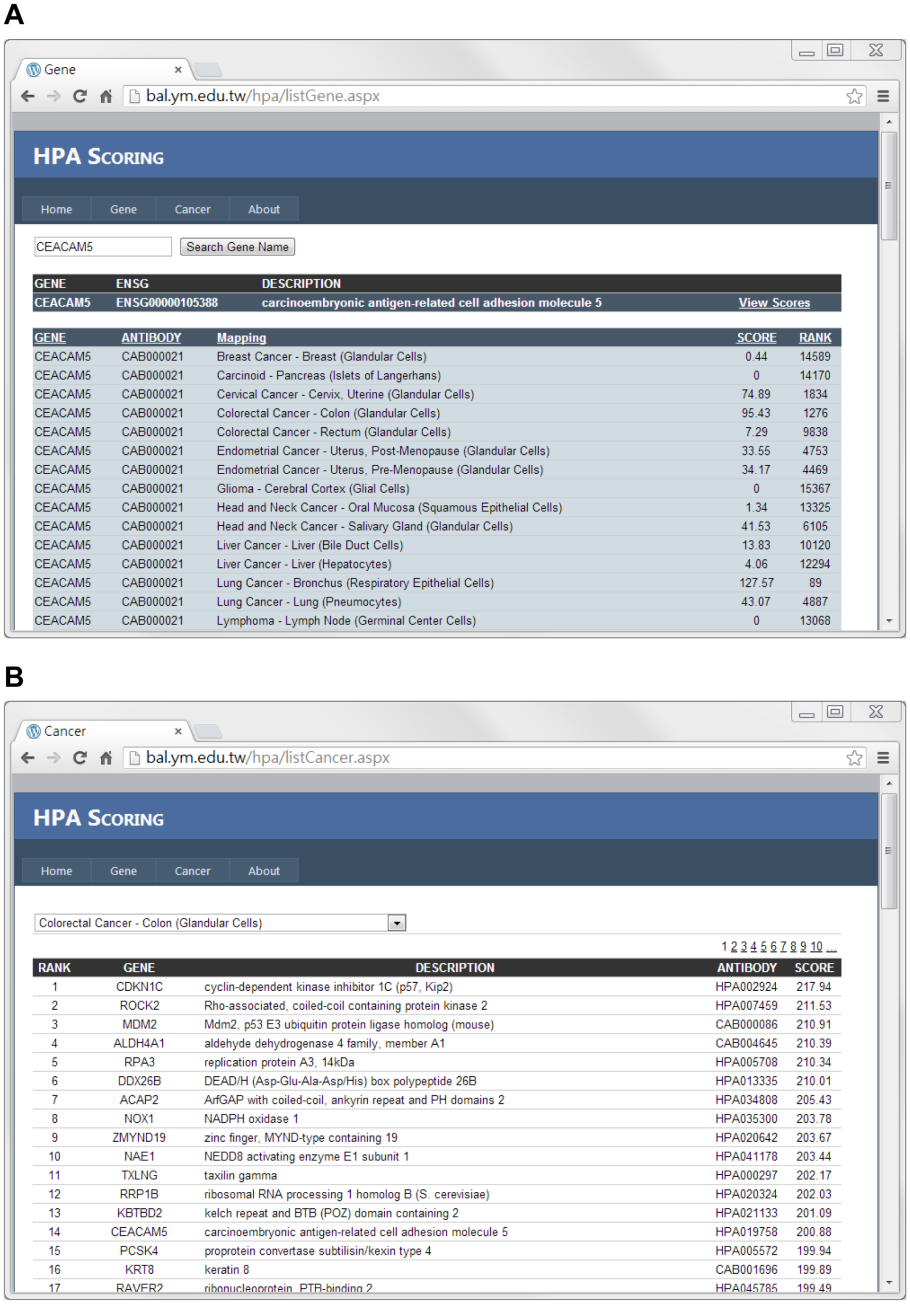
The HPA Scoring web server (http://bal.ym.edu.tw/hpa/). (A) The result of querying by gene name. (B) The result of querying by the mapping of a cancer type.

We have comprehensively calculated the scores for all the antibodies used in the HPA for each of the 27 mappings and this resulted in 457110 scores. Instead of summarizing these into a huge flat supplementary file, all the calculated scores are available on a web site that allows queries to be made (http://bal.ym.edu.tw/hpa/) (Figure 2). The web site, HPA Scoring, provides two query modes: a query by gene name and a query by cancer type. For a given gene name, HPA Scoring lists the score and rank of the antibodies used for each mapping (Figure 2A). For a given mapping of a cancer type, HPA Scoring reports a gene list, the entries in which are sorted by antibody score (Figure 2B). In the following part of the study, we carry out a verification of whether or not the proposed scoring approach is able to identify antibodies that satisfy the following criteria. Firstly, that the captured protein is overexpressed in the target cancer tissue, and, secondly, that the degree of the overexpression is significant and specific to the cancer. In the second part of this verification, we have also used colorectal cancer as the model disease and applied a method of cancer marker discovery specifically using our proposed scoring approach to the colorectal cancer dataset.

### The ability of the scoring approach to identify abundant proteins in cancer tissues

For each mapping, we select the top 100 antibodies according to their *Scores*, and perform a one-sample *t*-test in order to verify whether or not the average *EiC* of these 100 antibodies is statistical higher than that of all of the tested antibodies. The one-sample *t*-test is often used to measure the mean difference between a sample and a known population mean. We apply the one-sample *t*-test because we can determine the average *EiC* of all the tested antibodies, namely the population mean. The statistical significances of the *EiC* mean differences between the top100 antibodies and all the tested antibodies for each mapping are listed in Table 2. According to the *p*-values reported by the one-sample *t*-test, all the 27 *EiC* mean differences are statistical significant. The results of these tests demonstrate the ability of our scoring approach to identify abundant proteins in cancer tissues.

**Table 2:** The statistical significance of the *EiC* mean differences between the top 100 antibodies and all the tested antibodies.

	All the tested antibodies	Top 100 antibodies^1^		
Mapping ID	Mean	Mean	Standard deviation	*p*-value^2^
Breast	86.967	210.927	12.894	<0.001
Carcinoid	78.322	213.083	13.889	<0.001
Cervical-A	70.833	207.705	13.866	<0.001
Cervical-B	70.833	211.265	12.488	<0.001
Colorectal-A	95.679	210.295	12.865	<0.001
Colorectal-B	95.549	210.435	12.72	<0.001
Endometrial-A	76.765	205.513	13.47	<0.001
Endometrial-B	76.731	208.591	12.639	<0.001
Glioma	61.147	212.212	11.275	<0.001
Head & neck-A	83.165	218.938	9.166	<0.001
Head & neck-B	83.162	219.875	8.492	<0.001
Cholangio	86.078	222	5.871	<0.001
Hepato	75.282	211.352	13.172	<0.001
Lung-A	65.822	178.636	25.491	<0.001
Lung-B	66.014	207.776	10.676	<0.001
Lymphoma-A	53.113	200.519	15.218	<0.001
Lymphoma-B	53.113	202.852	14.863	<0.001
Melanoma	79.367	210.641	9.016	<0.001
Pancreatic	83.807	207.797	12.52	<0.001
Prostate	79.458	206.901	12.988	<0.001
Renal	59.437	200.705	15.034	<0.001
Skin	62.645	207.807	16.649	<0.001
Stomach-A	75.516	202.007	14.891	<0.001
Stomach-B	75.467	207.048	14.199	<0.001
Testis	75.369	210.936	11.128	<0.001
Thyroid	96.606	218.875	9.891	<0.001
Urothelial	77.656	194.347	17.655	<0.001

1 The 100 antibodies were selected on the basis of their *Scores*.

2 The *p*-values reported were obtained by one-sample *t*-test.

### The significance and cancer-specificity of the *ED* of top-ranked antibodies

In order to make sure the proposed scoring approach is capable of identifying proteins that are significantly overexpressed in cancer tissues, we perform a one-sample *t*-test to verify whether or not the average *ED* of the top 100 antibodies is statistical higher than that of all of the tested antibodies. The statistical significances of the *ED* mean differences between the top 100 antibodies and all the tested antibodies are listed in Table 3. According to the *p*-values reported by the one-sample *t*-test, all the 27 *ED* mean differences are statistical significant. The test results demonstrate the ability of our scoring approach to identify proteins that are highly expressed in the cancer of interest. Please note that the top 100 antibodies have an up-regulated trend (positive *ED* sample mean) for all the 27 mappings. This contrast with the results for most of the tested antibodies, which show a down-regulated trend in cancer tissues (22 out of the 27 mappings have a negative *ED* population mean).

**Table 3:** The statistical significance of the *ED* mean differences between the top 100 antibodies and all the tested antibodies.

	All the tested antibodies	Top 100 antibodies^1^		
Mapping ID	Mean	Mean	Standard deviation	*p*-value^2^
Breast	−11.035	97.927	37.331	<0.001
Carcinoid	−3.655	113.333	37.197	<0.001
Cervical-A	−13.116	125.455	45.193	<0.001
Cervical-B	−2.811	121.015	41.49	<0.001
Colorectal-A	−30.496	92.995	35.012	<0.001
Colorectal-B	−33.668	80.685	31.201	<0.001
Endometrial-A	−14.956	75.013	31.704	<0.001
Endometrial-B	−11.921	88.341	34.803	<0.001
Glioma	13.024	121.612	40.476	<0.001
Head & neck-A	4.52	143.588	41.322	<0.001
Head & neck-B	1.333	148.475	37.688	<0.001
Cholangio	37.899	185.75	32.201	<0.001
Hepato	−5.828	110.852	44.067	<0.001
Lung-A	−58.02	67.486	40.632	<0.001
Lung-B	13.475	145.776	37.781	<0.001
Lymphoma-A	−8.229	94.219	35.837	<0.001
Lymphoma-B	−11.273	88.602	31.782	<0.001
Melanoma	−0.421	162.141	39.514	<0.001
Pancreatic	−21.58	116.147	36.815	<0.001
Prostate	−13.088	88.051	33.274	<0.001
Renal	−59.582	77.754	36.312	<0.001
Skin	−12.319	93.407	38.835	<0.001
Stomach-A	−42.253	91.407	38.16	<0.001
Stomach-B	−44.785	94.598	40.061	<0.001
Testis	−28.357	105.686	34.966	<0.001
Thyroid	−15.33	107.125	44.123	<0.001
Urothelial	−36.808	68.547	36.583	<0.001

1 The 100 antibodies were selected on the basis of their *Scores*.

2 The *p*-values reported were obtained by one-sample *t*-test.

**Figure 3:**
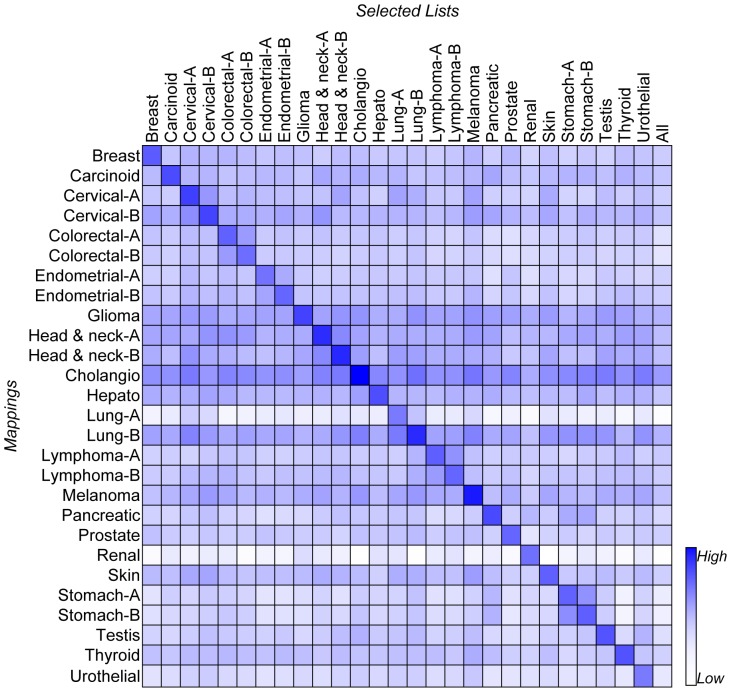
Specificity of the average *ED* of the top 100 antibodies selected for each mapping. In this heat map, large *ED* values are colored dark blue and small *ED* values are colored light blue. The entry (*i*, *j*) on the heat map represents the average *ED* of the top 100 antibodies of the *j*-th mapping calculated for the *i*-th mapping. The rightmost column, All, lists the average *ED* of all the tested antibodies calculated for each of the 27 mappings.

The top100 antibodies of each mapping were also used to verify whether or not the proposed scoring approach is capable of identifying proteins whose overexpression is specific to the cancer of interest. For the top 100 antibodies of a specific mapping, their average *ED* is determined for each of the 27 mappings. The obtained 27 *ED* means were then organized into a heat map with large *ED* values colored in dark blue and small *ED* values colored in light blue (Figure 3). The entry (*i*, *j*) in the heat map represents the average *ED* of the top 100 antibodies of the *j*-th mapping calculated for the *i*-th mapping. The rightmost column, All, lists the average *ED* values of all the tested antibodies calculated for each of the 27 mappings; namely the entries located within this column are population *ED* means. The heap map therefore has the dimensions 27 by 28. The dark blue entries located along the diagonal reveal that the average *ED* of the antibodies selected for a mapping are specific to that mapping. In contrast, most of the entries in the heap map have average *ED* for the antibodies selected of a mapping that are similar to the population *ED* mean if they are tested for another mapping. Every row in the heap map confirms the observation that for a certain mapping, the average *ED* values of the antibodies selected for this mapping are higher than that of antibodies selected for other mappings. Every column in the heat map also agrees with another observation, namely that for the 100 antibodies selected for a specific mapping, their average *ED* is only significant for selected mapping and is similar to the population mean for other mappings. The findings of this evaluation demonstrate that the *ED* of top-ranked antibodies is specific to the cancer of interest.

In summary, the proposed scoring approach shows great potential as a means of identifying abundant and cancer-specific proteins in tissues.

### Application of the approach to cancer marker discovery

In this section we use an evaluation cohort to demonstrate how the proposed scoring approach can be used to screen possible markers for cancers. The cohort consists of 1482 up-regulated membrane proteins from 28 patients who had been diagnosed with colorectal cancer [25]. We apply the following three filtering rules in order to select possible cancer markers from this cohort. Rules similar to the last two listed below have been widely used in biomarker discovery.

**Rule 1.** A protein with antibody score 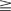
100 in either the colorectal-colon mapping or the colorectal-rectum mapping is selected.
**Rule 2.** An up-regulated protein with an average fold change 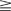
2 is selected.
**Rule 3.** An up-regulated protein with a fold change 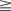
2 in more than 14 patients is selected.

The proteins selected by these criteria were then further analyzed using the *Biomarker Filter* provided by the IPA (Ingenuity Systems, http://www.ingenuity.com). Each protein with potential biomarker or disease application is annotated by the IPA during this process.

**Figure 4:**
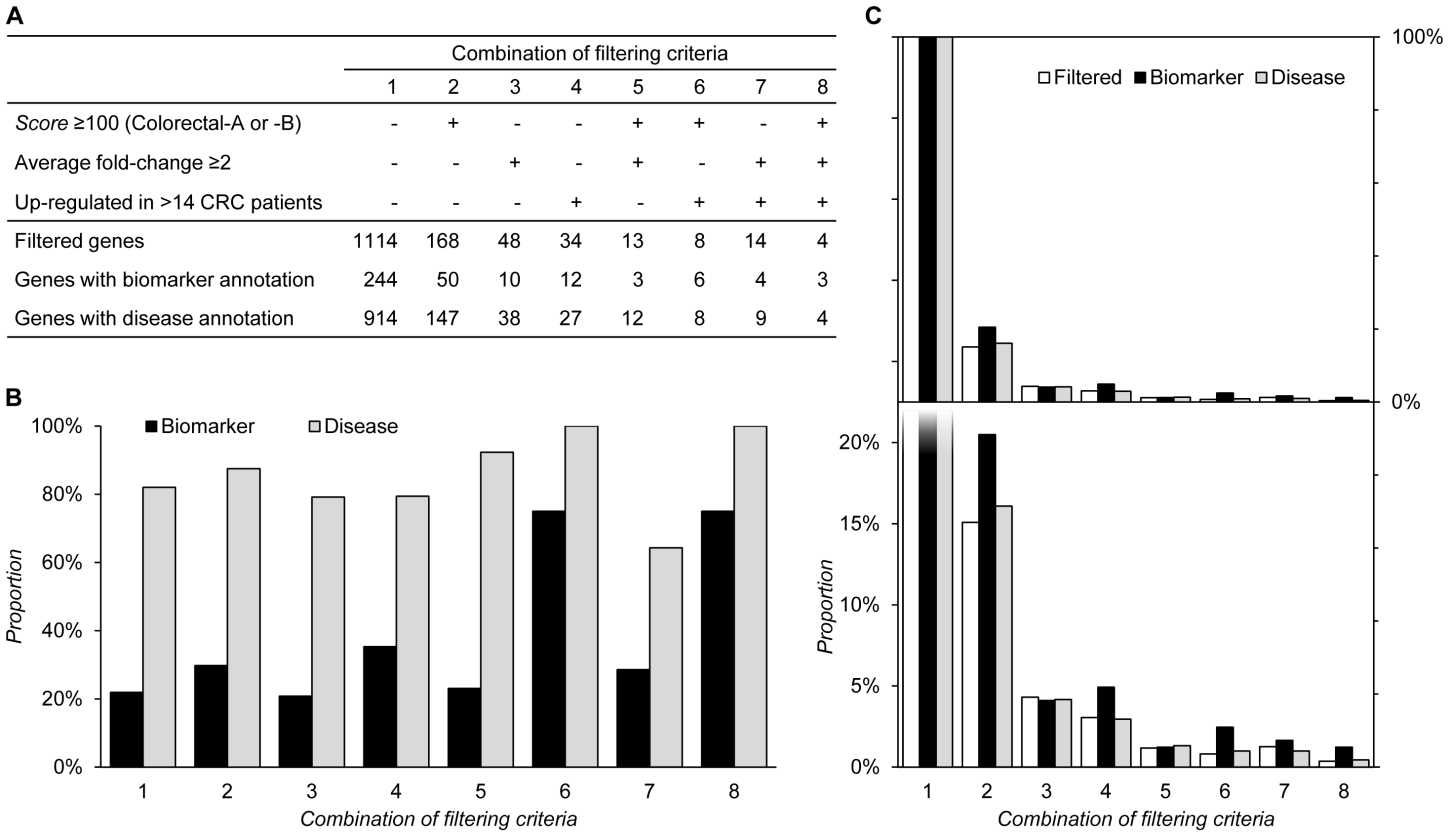
The results of various combinations of filtering criteria when applied to a cohort of 1482 membrane proteins. (A) The rules that are used to screen genes are marked with a plus sign and otherwise there is a minus sign. For each combination, the numbers of filtered genes, genes with biomarker annotation, and genes with disease annotation are listed. (B) The proportions of annotated biomarkers and disease-related genes to filtered genes of each combination are shown. (C) The proportion of the filtering results to our sample population is shown. This figure is a panel chart that has two panels; the upper one has an axis that covers the full range of data, while the lower one has an axis that focuses on data within the range 0%–25%.

Eight combinations of filtering criteria were evaluated. Each of the combinations takes into consideration different combinations of the various filtering rules. The filtering results are shown in Figure 4. Those rules that are used to screen genes are marked a plus sign in Figure 4A and otherwise they are marked with a minus sign. For each combination, the numbers of filtered genes, genes with biomarker annotation, and genes with disease annotation are also listed in Figure 4A. Special attention should be paid to Combination 1. In this combination we simply match all of the 1482 proteins against the HPA version10.0 to see how many related genes are indexed in the HPA; specifically, no explicit filtering rules are applied to select possible markers. There are 1114 indexed genes, among which 244 genes have biomarker annotation and 914 genes have disease annotation from the IPA. The result of Combination 1 forms our sample population. The proportions of the annotated biomarkers and disease-related genes to the filtered genes of each combination are shown in Figure 4B. The proportion of the filtering results to our sample population is shown in Figure 4C. Namely, the proportions of the filtered genes to all the 1114 indexed genes, the filtered biomarkers to the 244 annotated markers, and the filtered disease-related genes to the 914 annotated disease-related genes; these are listed in Figure 4C. Figure 4C is a panel chart that has two panels; the upper one has an axis that covers the full range of data, while the lower one has an axis that focuses on the data within the range 0%–25%.

We then applied Combinations 2, 3, and 4 to evaluate the effect of Rule 1, Rule 2, and Rule 3, respectively. Combination 2, namely Rule 1 alone, allowed a certain degree of success in biomarker discovery; the proportion of the annotated biomarkers to the filtered genes is increased from 21.9% to 29.8% (Figure 4B). Moreover, Combination 2 has the ability to screen disease-related genes and the proportion of the annotated disease-related genes to the filtered genes is increased from 82.0% to 87.5% (Figure 4B). Applying Combination 2 shrinks the sample size to 15.1% but keeps 20.5% of the annotated biomarkers and 16.1% of the annotated disease-related genes (Figure 4C). Applying Combination 3, namely Rule 2 alone, evenly shrinks the sample size, annotated biomarkers, and annotated disease-related genes (4.3%, 4.1%, 4.2%, Figure 4C). The proportion of the annotated biomarkers and disease-related genes to the filtered genes is also kept at the same level as those in the sample population (20.8% *vs*. 21.9%; 79.2 *vs*. 82.0%, Figure 4B). The effect of applying Combination 3 is somewhat like random sampling. Combination 4, namely Rule 3 alone, has best biomarker screening ability among the three filtering rules; the proportion of the annotated biomarkers to the filtered genes is increased from 21.9% to 35.3% (Figure 4B). Applying Combination 4 evenly shrinks the sample size and annotated disease-related genes (3.1% and 3.0%) but keeps 4.9% of the annotated biomarkers (Figure 4C). It seems that applying Rules 1 and 3 are both effective strategies when performing biomarker discovery.

We also evaluate the performance of combinations that use two filtering rules together. Combination 5 applies Rules 1 and 2, Combination 6 applies Rules 1 and 3, and Combination 7 applies Rules 2 and 3. All the three combinations dramatically shrink the sample size to a scale that is suitable for wet-lab validation; applying Combinations 5, 6, and 7 generates 13, 8, and 14 filtered genes, respectively (Figure 4A). Combination 6 retains the largest portion of biomarkers. The proportion of annotated biomarkers to filtered genes is increased from 21.9% to 75% (Figure 4B). Combinations 5 and 7 produce similar results in terms of identifying annotated biomarkers, while Combination 5 has a better disease-related gene screening ability. The proportion of the annotated disease-related genes to the filtered genes is 92.3% when applying Combination 5 but only 64.3% when applying Combination 7 (Figure 4B). The evaluation results agree with our observation that Rule 1 in combination with Rule 3 is able to effectively screen potential biomarkers. Rule 1 in combination with Rule 2 or Rule 2 in combination with Rule 3 also improves the ability to screen biomarkers or disease-related genes, but is less powerful than the combination of Rules 1 and 3. The results of Combinations 5 and 7 agree with the observation that Rule 3 is good at identifying biomarkers while Rule 1 is good at identifying disease-related genes. In this evaluation, the filtering performance is dominated by finding proteins that are overexpressed in most patients and the proposed scoring mechanism indeed does seem to play an important role. Interesting, viewing Figure 4, those proteins that show a significant but average fold change in patients may not be good biomarker candidates. Such a protein may only be highly expressed in a small portion of patients, but is normally expressed in most patients.

Finally, we apply Combination 8 that combines all the three rules to select potential biomarkers for colorectal cancer. This approach identified four filtered genes, among which three genes have biomarker annotation and all the four genes have disease-related annotation from the IPA. Information on the four proteins is listed in Table 4. Two of the genes, Carcinoembryonic antigen-related cell adhesion molecule 5 and Carcinoembryonic antigen-related cell adhesion molecule 6, CEACAM5 and CEACAM6, respectively, belong to the carcinoembryonic antigen (CEA) family. CEA family protein have been found to be increased in sera of patients with breast cancer, lung cancer, gastric cancer, pancreatic cancer, bladder cancer, medullary thyroid cancer, head and neck cancer, cervical cancer, hepatic cancer, lymphoma, and melanoma [30]. Nevertheless, since CEA was first found to be elevated in sera of patients with colorectal cancer in 1969, it has been used as a colorectal cancer marker for more than 40 years [26], [31]–[33]. Currently CEA is the most widely used marker for colorectal cancer; it is primarily used for prognostic monitoring [26]. If we consider the other two proteins, firstly, Cathelicidin antimicrobial peptide, CAMP (also known as LL-37), has several functions, including cell chemotaxis, immune mediator induction, inflammatory response regulation, and antimicrobial activity [34]. Recent studies have pinpointed an emerging role for CAMP in cancer. Although the function role of CAMP in cancer development remains unclear, CAMP has been associated with tumor cell proliferation, survival, and metastasis, and these findings have indicated its therapeutic application potential [35]. In addition to the direct effects of cathelicidin on tumor epithelium, cathelicidin may promote tumor growth through alternative mechanisms [36] and is overexpressed in breast cancer [37],[38], lung cancer [39], prostate cancer [40], and ovarian cancer [41]. In contrast, cathelicidin exhibits tumor-suppressing effects in gastric cancer [42], acute myeloid [43], and lymphocytic leukemia [44]. A recent study has further shown that cathelicidin may induce apoptosis through an alternative caspase-independent pathway in colon cancer, suggesting a tumor-suppressing mechanism for cathelicidin in colon tumorigenesis [27]. Patients with lung cancer have been found to have increased serum levels of cathelicidin, suggesting it has a potential role as a marker identifying cancer progression [36]. Secondly, Annexin A4 (ANXA4) is a member of the annexin family of calcium-dependent phospholipid binding proteins that binds to certain membrane phospholipids in a Ca(2+)-dependent manner [45]. Overexpression of ANXA4 has been associated with prostate cancer [46], pancreatic adenocarcinoma [47], renal clear cell carcinoma [48], colorectal carcinoma [28], [29], gastric cancer [49], and ovarian carcinoma [50], [51]. Although ANXA4 does not have biomarker annotation from the IPA, recent studies have suggested it may be a potential biomarker candidate for gastric cancer and colorectal cancer [52], [28].

**Table 4:** The four filtered genes obtained by applying Combination 8.

Gene	Fold Change	Number of Patients	HPA *Score*	IPA annotated Biomarker
CEACAM5	6.41	24	200.5	Yes
CEACAM6	4.32	21	155.2	Yes
ANXA4	2.07	15	138.84	No
CAMP	4.29	20	132.92	Yes

Taking the above findings as a whole, all the four identified filtered genes have experimental evidence that supports their potential as biomarkers for colorectal cancer. The filtering results of this model disease suggest that the proposed scoring approach based on the IHC annotation provided by the HPA is an effective approach. Even though the HPA has received criticism based on the unreliable quality of the IHC images and antibodies used, our proposed score appeared to provide useful additional information that assists the filtering of cancer marker candidates obtained from high-throughput omics experiments. As the antibody and IHC imaging data are continuously being improved and optimized through the efforts of the HPA, we believe that the reliability issue can be gradually resolved in the future.

## Supporting Information

Table S1 Expression fold change of 1482 proteins identified from 28 colorectal patients
(XLS)

Table S2 Clinical information of the 28 patients in colorectal cancer cohort
(XLS)
